# Emergence of Tigecycline‐Resistant *Pseudomonas aeruginosa* Harbouring *tmexC6D6*‐*toprJ1b* From Hospital Sewage in Japan

**DOI:** 10.1111/1758-2229.70275

**Published:** 2026-03-03

**Authors:** Shotaro Maehana, Masato Suzuki, Naoko Ishimura, Hiroki Izawa, Ryotaro Eda, Masaki Nakamura, Mohan Amarasiri, Takashi Furukawa, Fumiaki Kojima, Kazunari Sei, Makoto Kubo

**Affiliations:** ^1^ Department of Microbiology School of Allied Health Sciences, Kitasato University Kanagawa Japan; ^2^ Department of Environmental Microbiology Graduate School of Medical Sciences, Kitasato University Kanagawa Japan; ^3^ Regenerative Medicine and Cell Design Research Facility Kanagawa Japan; ^4^ Antimicrobial Resistance Research Center, Japan Institute for Health Security Tokyo Japan; ^5^ Department of Clinical Laboratory Kitasato University Hospital Kanagawa Japan; ^6^ Research Center for Biosafety, Japan Institute for Health Security Tokyo Japan; ^7^ Department of Laboratory Medicine School of Medicine, Kitasato University Kanagawa Japan; ^8^ Environmental Water Quality Engineering Laboratory, Department of Civil and Environmental Engineering Graduate School of Engineering, Tohoku University Miyagi Japan; ^9^ Department of Environmental Hygiene School of Allied Health Sciences, Kitasato University Kanagawa Japan; ^10^ Department of Pharmacology School of Allied Health Sciences, Kitasato University Kanagawa Japan

**Keywords:** hospital sewage, Japan, *Pseudomonas aeruginosa*, tigecycline resistance, *tmexC6D6‐toprJ1b*

## Abstract

The mobile *tmexCD‐toprJ* gene clusters encode resistance‐nodulation‐division (RND)‐type multidrug efflux pumps which confer resistance to multiple antimicrobials, including tigecycline. Here we report the first identification of *tmexCD‐toprJ‐*harbouring *Pseudomonas aeruginosa* strain KAM950, isolated from hospital sewage in Japan in 2022. The isolate exhibited reduced susceptibility to tigecycline and carbapenems. Complete genome sequence analysis showed that KAM950 belongs to sequence type 244 (ST244) according to multilocus sequence typing, an internationally recognised epidemic clone, and harbours multiple antimicrobial resistance genes, including the *tmexCD‐toprJ* variant, *tmexC6D6‐toprJ1b*. Notably, the *tmexC6D6‐toprJ1b* gene cluster was located on the chromosome, adjacent to the transcriptional regulator gene *tnfxB6* and an IS*5*/IS*1182* family transposase gene. Furthermore, an IS*4*‐mediated disruption of the porin gene *oprD* was observed, potentially contributing to carbapenem resistance. BLASTn analysis revealed that the IS*5*/IS*1182‐tnfxB6‐tmexC6D6‐toprJ1b* gene cluster present in both chromosomal and plasmid sequences among the order *Pseudomonadaceae*, indicating potential horizontal gene transfer of *tnfxB6‐tmexC6D6‐toprJ1b* mediated by IS*5*/IS*1182*. Our findings highlight the ongoing expansion of variant diversity and geographic spread of *tmexCD‐toprJ*‐like gene clusters, and underscore the importance of genomic surveillance for emerging antimicrobial resistance determinants in both clinical and environmental settings.

## Graphical abstract

This study isolated a *Pseudomonas aeruginosa* strain (KAM950) carrying the *tmexCD‐toprJ* gene cluster from hospital sewage in Japan. The strain exhibited reduced susceptibility to tigecycline and carbapenems, harboured chromosomal *tmexC6D6‐toprJ1b* and *oprD* disruption, and belonged to epidemic clone ST244, emphasising emerging multidrug resistance and the need for genomic surveillance.

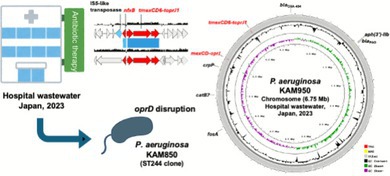

## 1 Introduction

A third‐generation tetracycline of the glycylcycline class, tigecycline was developed in the 1990s from minocycline to overcome tetracycline resistance (Zhanel et al. 2004). It exhibits broad‐spectrum activity against both gram‐positive and gram‐negative bacteria, including strains resistant to multiple clinically important antimicrobial classes, such as aminoglycosides, fluoroquinolones, and carbapenems, as well as tetracyclines. Employed as a salvage therapy for severe infections caused by carbapenem‐ and colistin‐resistant bacteria (Tasina et al. 2011), tigecycline demonstrates particular efficacy against extensively drug‐resistant *Enterobacterales* and *Acinetobacter* species (Brust et al. 2014). The dissemination of tigecycline resistance poses a global public health threat to treatment options. Resistance‐nodulation‐cell division (RND)‐type multidrug efflux pumps, such as MexCD‐OprJ, confer resistance to multiple antimicrobials (Du et al. 2018). The transferrable *tmexCD‐toprJ* gene clusters, which encode the RND‐type multidrug efflux pump TMexCD‐TOprJ, confer multidrug resistance, including resistance to tetracyclines and tigecycline. These gene clusters are believed to have originated from the chromosomal *mexCD‐oprJ* efflux pump gene cluster homologue in *Pseudomonas* species (Lv et al. 2020; Peng et al. 2024).

*tmexCD‐toprJ* gene clusters have been identified in gram‐negative bacteria from humans, animals, and various environmental sources, and have globally dispersed via mobile genetic elements, such as plasmids (Anyanwu et al. 2022). Most variants of *tmexCD‐toprJ*, including *tmexCD1‐toprJ1*, have been reported mainly in *Enterobacterales* (Anyanwu et al. 2022), whereas the *tmexC6D6‐toprJ1b* variant appears to be primarily associated with *Pseudomonas* species (Wang et al. 2023). To date, only two *Klebsiella pneumoniae* clinical isolates that harbour the *tmexCD1‐toprJ1* gene cluster on the IncFIB(K) and the IncHI1B/IncFIB(K) plasmid, respectively, have been reported in Japan (Hirabayashi et al. 2025). Although the clinical use of tigecycline remains limited and the prevalence of *tmexCD‐toprJ* gene clusters is presumed to be extremely low in Japan, no studies have yet investigated their presence in non‐clinical settings.

This study aimed to investigate tigecycline‐resistant and multidrug‐resistant bacteria in hospital sewage in Japan as part of a One Health approach (Martinengo et al. 2025; GBD 2021). We identified a *Pseudomonas aeruginosa* isolate KAM950 harbouring *tmexC6D6‐toprJ1b* and conducted genomic and molecular characterisation.

## 2 Experimental Procedures

### 2.1 Bacterial Isolation and Identification

In 2022, screening for carbapenem‐ and tigecycline‐resistant bacteria was conducted using sewage water collected from a tertiary emergency hospital in Kanagawa Prefecture, Japan. An aliquot (0.1 mL) of the sample was pre‐cultured overnight at 37°C in MacConkey broth supplemented with 8 mg/L meropenem and 8 mg/L tigecycline. Bacterial isolates were subsequently obtained by plating on desoxycholate‐hydrogensulfide‐lactose (DHL) agar containing 8 mg/L meropenem and 8 mg/L tigecycline and incubated overnight at 37°C. A total of 90 isolates were confirmed as *P. aeruginosa* by the MALDI Biotyper system (Bruker), with isolate KAM950 selected for detailed analysis.

### 2.2 Antimicrobial Susceptibility Testing

Antimicrobial susceptibility testing (AST) for KAM950 was performed by the agar dilution method, following the Clinical and Laboratory Standards Institute (CLSI) guidelines (CLSI 2025). Briefly, antimicrobial solutions were prepared in two‐fold serial dilutions and incorporated into cation adjusted Mueller–Hinton agar (BD) using *Escherichia coli* ATCC 25922 as the control. Upon solidification, bacterial suspensions standardised to approximately 10^4^ CFU per spot were applied using a micro‐planter MIT‐60 (Sakuma Seisakujo). Plates were incubated at 35°C for 16–20 h under appropriate atmospheric conditions. The MIC was defined as the lowest concentration showing no visible growth. The susceptibility of the strain to antibiotics was assessed multiple times on different days. CLSI breakpoints for gentamicin, minocycline, and tigecycline were not available for *Pseudomonas* species.

### 2.3 Whole‐Genome Sequencing

Whole‐genome sequencing of KAM950 was performed using both the NovaSeq X platform (Illumina) and the MinION platform (Oxford Nanopore Technologies) equipped with an R10.4.1 flow cell. The Illumina sequencing library (paired‐end, insert size 500–900 bp) was prepared using the Nextera XT DNA Library prep kit, while the ONT sequencing library was prepared using the rapid barcoding kit (SQK‐RBK114.24). Basecalling of ONT reads was performed using Dorado v0.5.3 with the high‐accuracy model.

### 2.4 Genome Assembly and Annotation

Hybrid assembly of ONT and Illumina reads was performed de novo using Unicycler v0.5.0 (https://github.com/fenderglass/Flye) with the default parameters, resulting in a complete circular chromosome. Gene annotation was performed using the DFAST server (https://dfast.nig.ac.jp) with the default parameters.

### 2.5 Genomic Analysis

Average nucleotide identity (ANI) analysis was performed using the DFAST server (https://dfast.nig.ac.jp). MLST analysis was performed using mlst v2.16.1 (https://github.com/tseemann/mlst) with the *P. aeruginosa* species‐specific scheme from PubMLST, as originally developed by Curran et al. (2004). Antimicrobial resistance genes (ARGs) were identified using ResFinder v4.5.0 with the default parameters via the Center for Genomic Epidemiology (CGE) server (http://www.genomicepidemiology.org). Linear comparisons of 5‐kb upstream and downstream regions of *tmexCD‐toprJ* and *mexCD‐oprJ* were performed using BLASTn and visualised with Easyfig v2.2.2 (http://mjsull.github.io/Easyfig/).

## 3 Results and Discussion

A *P. aeruginosa* isolate, KAM950, exhibiting resistance to both carbapenems and tigecycline, was isolated from hospital sewage in Japan in 2022. Carbapenem‐resistant *P. aeruginosa* , one of clinically significant antimicrobial resistant bacterial pathogens, is included in the WHO priority pathogens list (Sati et al. 2025).

AST revealed that KAM950 exhibited resistant‐level MICs to piperacillin (> 256 mg/L), piperacillin/tazobactam (128 mg/L), imipenem (16 mg/L), and meropenem (32 mg/L), along with elevated MICs for gentamicin (128 mg/L), minocycline (128 mg/L), and tigecycline (128 mg/L). For comparison, the reference strain *P. aeruginosa* PAO1 exhibits MICs of 1 mg/L for imipenem and 16 mg/L for tigecycline. Thus, the MICs of KAM950 increased approximately 16‐fold for imipenem and 8‐fold for tigecycline compared with PAO1, indicating a marked reduction in susceptibility to these agents.

Whole‐genome sequencing analysis using both short‐ and long‐read sequencers resulted in the complete genome of KAM950 consisting of a 6.7‐Mb chromosome (accession no. AP040125). KAM950 was confirmed as *P. aeruginosa* based on ANI showing 97.38% identity with *P. aeruginosa* NBRC12689^T^. According to multilocus sequence typing, KAM950 was classified as sequence type 244 (ST244), one of the global epidemic high‐risk clones associated with multidrug resistance, including carbapenem resistance, of *P. aeruginosa* (Del Barrio‐Tofiño et al. 2020).

**FIGURE 1:**
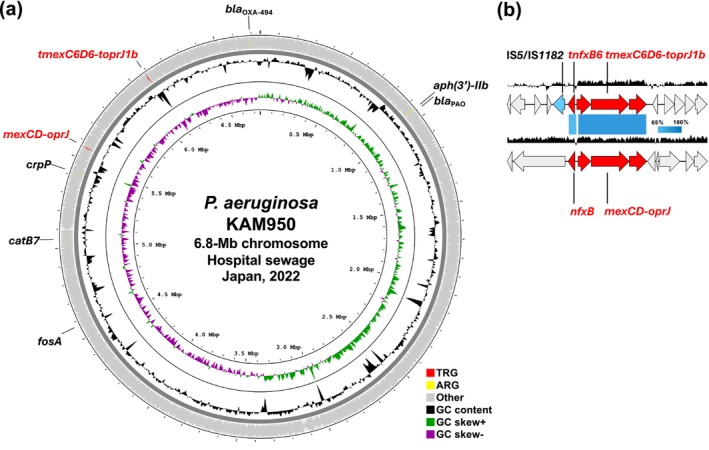
(a) Genomic structure of the *P. aeruginosa* KAM950 chromosome (accession no. AP040125) harbouring *tmexC6D6‐toprJ1b*, isolated from hospital sewage in Japan in 2022. Tigecycline resistance genes (TRG), other antimicrobial resistance genes (ARG), other coding sequences (CDS), mobile genetic elements (MGE), GC content, GC skew+, and GC skew– are highlighted by red, yellow, grey, light blue, black, green, and purple, respectively. (b) Structural comparison between the chromosomal regions surrounding *tnfxB6‐tmexC6D6‐toprJ1b* and *nfxB‐mexCD‐oprJ* gene clusters. Synteny blocks represent the indicated sequence identity.

KAM950 harboured multiple ARGs on the chromosome, including *aph(3′)‐IIb* (aminoglycoside resistance), *bla*_OXA‐494_ and *bla*_PAO_ (β‐lactam resistance), *catB7* (chloramphenicol resistance), *fosA* (fosfomycin resistance), *crpP* (fluoroquinolone resistance), and *tmexC6D6‐toprJ1b* (multidrug resistance including tetracycline and tigecycline resistance), the *tmexCD‐toprJ* variant first described in China in 2023 (Wang et al. 2023), in addition to the chromosomal *mexCD‐oprJ* (Figure 1a). Furthermore, an insertion sequence belonging to the IS*4* family was found integrated into the porin gene *oprD*, potentially contributing to carbapenem resistance through porin loss (Wang et al. 2025).

**FIGURE 2:**
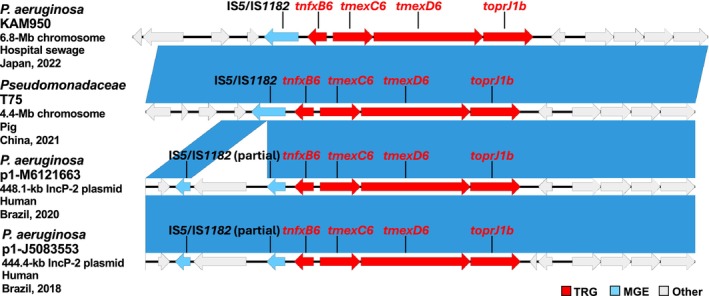
Structural comparison between the indicated *tmexC6D6‐toprJ1b*–containing regions in the *P. aeruginosa* KAM950 chromosome (accession no. AP040125) in this study, the *Pseudomonadaceae* bacterium T75 chromosome (accession no. CP113226), the *P. aeruginosa* M6121663 plasmid p1‐M6121663 (accession no. CP166820) and the *P. aeruginosa* J5083553 plasmid p1‐J5083553 (accession no. CP166820). The tigecycline resistance gene (TRG), mobile gene elements (MGE), and other coding sequences (Other) are highlighted by red, light blue, and grey, respectively. Synteny blocks represent 100% sequence identity.

The *tmexC6D6‐toprJ1b* gene cluster was encoded at a different location from *mexCD‐oprJ* (Figure 1a) and was accompanied by the adjacent regulatory gene *tnfxB6* (Figure 1b). Notably, an IS*5*/IS*1182* family transposase gene was identified upstream of *tnfxB6‐tmexC6D6‐toprJ1b*, suggesting the potential for the IS*5*/IS*1182*‐mediated horizontal gene transfer of this gene cluster (Figure 1b). A BLASTn analysis of the chromosomal IS*5*/IS*1182*‐*tnfxB6‐tmexC6D6‐toprJ1b*‐containing region of KAM950 using the NCBI nr database revealed an identical sequence in the chromosome of *Pseudomonadaceae* strain T75, from a pig in China in 2021 (Figure 2), and highly similar sequences in *P. aeruginosa* IncP‐2 megaplasmids p1‐M6121663 and p1‐J5083553, from humans in Brazil in 2020 and 2018, respectively (Figure 2). Indeed, IncP‐2 megaplasmids have been shown to be associated with clinically important ARGs, including *tmexCD‐toprJ (*Shintani et al. 2022 *)*.

This study reports the first identification and characterisation of the *tmexC6D6‐toprJ1b* variant in Japan. *tmexC6D6‐toprJ1b* may be spreading globally, occasionally through IS*5*/IS*1182*‐mediated transposition, integrating into both chromosomes and plasmids particularity of *Pseudomonadaceae*. The acquisition of *tmexCD‐toprJ* gene clusters by *P. aeruginosa* will likely enhance its intrinsic multidrug resistance, posing a clinical threat and necessitating heightened vigilance and continuous surveillance.

## Author Contributions

**Hiroki Izawa:** investigation, writing – review and editing. **Masaki Nakamura:** investigation, writing – review and editing. **Shotaro Maehana:** conceptualization, methodology, data curation, formal analysis, visualization, writing – original draft, writing – review and editing, project administration, supervision, investigation, validation, funding acquisition, resources. **Kazunari Sei:** writing – review and editing, resources, funding acquisition. **Fumiaki Kojima:** investigation, writing – review and editing, resources. **Mohan Amarasiri:** investigation, writing – review and editing. **Masato Suzuki:** conceptualization, data curation, formal analysis, visualization, writing – original draft, writing – review and editing, project administration, supervision, investigation, methodology, software, validation, funding acquisition, resources.

## Funding

This work was supported by the Japan Agency for Medical Research and Development (JP24fk0108665, JP24fk0108683, JP24fk0108712, JP24fk0108642, JP24gm1610003, JP24wm0225029, JP24wm0225022), the Ministry of Education, Culture, Sports, Science and Technology (JP22K17354, JP23K26235, JP23H00536, JP23K06556, JP22KK0058, JP25K13531), and the Environmental Restoration and Conservation Agency (JPMEERF25S21220, JPMEERF25S21212).

## Ethics Statement

The authors have nothing to report.

## Conflicts of Interest

The authors declare no conflicts of interest.

## Data Availability

The data that support the findings of this study are available from the corresponding author upon reasonable request. The data obtained by sequencing that support the findings of this study are openly available in GenBank/EMBL/DDBJ under the accession number AP040125.
